# From Glucose to Green Chemistry: Breakthrough in Microbial Production of Tartaric Semialdehyde

**DOI:** 10.1111/1751-7915.70149

**Published:** 2025-04-23

**Authors:** Shuangxi Li, Lingcheng Li, Qiwu Jiang, Jianfeng Wang, Xiaoming Sun, Liangliang Zhang, Jianfeng Yuan

**Affiliations:** ^1^ Xingzhi College Zhejiang Normal University Lanxi China; ^2^ Key Laboratory of Wildlife Biotechnology and Conservation and Utilization of Zhejiang Province Zhejiang Normal University Jinhua China; ^3^ School of Pharmacy China Pharmaceutical University Nanjing Jiangsu China

**Keywords:** *Gluconobacter oxydans*, modular assembly, tartaric semialdehyde, whole‐cell oxidation

## Abstract

L‐(+)‐tartaric acid (L‐TA) is a crucial hydroxy carboxylic chelator with extensive applications in the food and pharmaceutical industries. The synthesis of L‐TA from renewable biomass presents a promising approach to mitigating environmental impact and advancing green energy initiatives. Previous studies revealed that a mutant transketolase (TKTA_M) could catalyse the production of tartaric semialdehyde, a precursor to L‐TA. This study focuses on the development of a *Gluconobacter oxydans* cell factory for tartaric semialdehyde production, employing a combination of metabolic engineering and a modular strategy. The genetically modified *G. oxydans* T strain exhibited robust expression of the *tkt*A_M gene. The optimal pH and temperature for this strain were determined to be 6.0°C and 30°C, respectively. Under these conditions, the strain produced 32.21 ± 0.74 g/L of tartaric semialdehyde from glucose. Implementation of a “Push‐Pull” strategy enhanced tartaric semialdehyde production, resulting in a 23.85% increase in the *G. oxydans* T02 cell growth. In CSLP medium with 100 g/L glucose, the fermentation process yielded 48.88 ± 2.16 g/L of tartaric semialdehyde and 7.72 ± 1.56 g/L of residual 5‐KGA after 48 h. This resulted in a tartaric semialdehyde productivity rate of 1.018 g/L·h, representing an 87.82% improvement over flask fermentation. This study demonstrates a straightforward and efficient microbial process for the oxidation of glucose to tartaric semialdehyde, indicating its potential for industrial‐scale production and facilitating the synthesis of L‐TA from renewable resources.

## Graphical abstract

L‐(+)‐tartaric acid has extensive applications in many industries. It is formed via a chemical synthesis–enzyme catalysis approach utilising petroleum‐derived C4 lysate. In this study, we employ a combination of metabolic engineering and a modular strategy (“Push‐Pull”) to develop an engineered strain of *Gluconobacter oxydans* capable of producing tartaric semialdehyde from glucose, which is a promising candidate to synthesize L‐(+)‐tartaric acid.

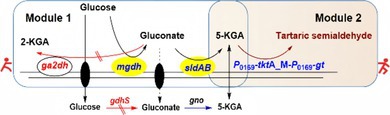

## 1 Introduction

The global shift toward sustainable development has intensified the search for alternatives to petrochemical‐based production processes, driven by environmental concerns and the imperative for carbon neutrality (Holechek et al. 2022; Saraf et al. 2023). Among these efforts, the biosynthesis of high‐value chemicals from renewable feedstocks has emerged as a promising strategy to reduce reliance on fossil resources while meeting industrial demands (Pyo et al. 2022). L‐(+)‐tartaric acid (L‐TA), a multifunctional hydroxy carboxylic acid, is widely used in the food, pharmaceutical, and textile industries (Su et al. 2024). L‐TA has seen annual demand surge to ~200,000 tons, with a growth rate exceeding 10% (Jin et al. 2020). Its industrial importance is comparable to that of other essential organic acids, including citric, lactic and malic acids.

Currently, industrial L‐TA production relies on a chemical synthesis‐enzyme catalysis hybrid process using petroleum‐derived C4 feedstocks (Scheme S1A). In this method, *cis*‐epoxysuccinate hydrolase (CESH, EC 3.3.2.3) selectively hydrolyzes disodium epoxysuccinate to yield L‐TA (Bao et al. 2017, 2020). However, this approach faces critical challenges, including volatility in petroleum prices, enzyme instability, and environmental pollution from byproducts (Li et al. 2014). China, a major L‐TA producer, grapples with these limitations, underscoring the need for sustainable alternatives.

Recent advances propose a biological oxidation‐chemical catalysis route (Scheme S1B), wherein 5‐keto‐D‐gluconic acid (5‐KGA) serves as a key intermediate. Metabolic engineering of microbial strains (e.g., *Gluconobacter oxydans* , *Escherichia coli* ) has achieved 5‐KGA titers of 162–179 g/L (Merfort et al. 2006a, 2006b; Tan et al. 2014; Yuan et al. 2016a, 2016b; Kataoka et al. 2022). Subsequently, chemical catalysis of 5‐KGA to L‐TA employs vanadate, palladium carbon, or transition metals (Matzerath et al. 1995; Hoshino et al. 2011; Yuan et al. 2016c). Yet, industrial adoption remains elusive due to low conversion efficiencies and catalyst costs.

A fully biological route for L‐TA synthesis is hindered by the absence of complete pathways in natural microorganisms. Although plants like *Vitis* and *Pelargonium* possess L‐TA biosynthetic genes (e.g., L‐idonate dehydrogenase, *IdnDH*, 2‐keto‐L‐gulonate reductase, *Vv2KGR*; DeBolt et al. 2006; Jia et al. 2019), the lack of L‐TA accumulation in model systems complicates gene discovery (Burbidge et al. 2021). Notably, the enzymatic cleavage of 5‐KGA's C4‐C5 bond, a proposed step (Figure S1) toward L‐TA via transketolase (*TK*) and tartaric semialdehyde dehydrogenase (*TSAD*), remains enigmatic. Early work by Salusjärvi et al. (2004) suggested *TK* and succinate semialdehyde dehydrogenase convert 5‐KGA to L‐TA in *E. coli* , but mechanistic validation is lacking. Recently, Wang et al. (2022) engineered a *TK* variant (Gene ID: 947420, TKTA_M, R358I/H461S/R520Q) with 55.3% enhanced activity toward 5‐KGA, offering preliminary evidence for tartaric semialdehyde as a precursor. However, optimising this pathway in vivo for industrial‐scale production remains unresolved.

**FIGURE 1:**
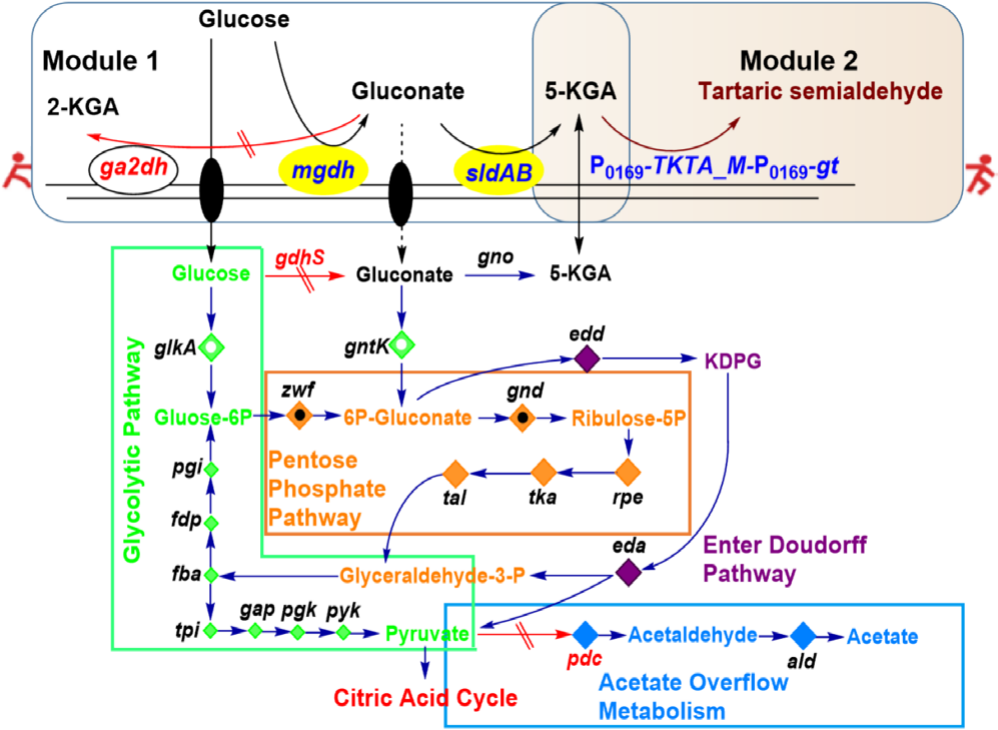
Schematic representation of the metabolic pathway of the engineered *G. oxydans* strain for tartaric semialdehyde biosynthesis. Module 1, the “Push” module is that promote the 5‐KGA supply; Module 2, the “Pull” module is that boost the tartaric semialdehyde biosynthesis; green box, glycolytic pathway; blue box, acetate overflow metabolism; brown box, pentose phosphate pathway; red double slash arrow, cut off the pathway.

*Gluconobacter oxydans* , a gram‐negative bacterium with exceptional oxidative capacity (Mao et al. 2023), presents an ideal chassis for L‐TA precursor synthesis. Its membrane‐bound dehydrogenases enable stereoselective oxidation of sugars to valuable ketones and acids (la China et al. 2018; Hua et al. 2020), albeit with inherent metabolic inefficiencies (Yuan et al. 2016a). Here, the *G. oxydans* strain was engineered to over‐express *tktA*_M gene, employing a “Push‐Pull” strategy (Figure 1), wherein 5‐KGA flux is augmented (“Push”) while tartaric semialdehyde synthesis is enhanced (“Pull”). This dual approach aims to overcome pathway bottlenecks, reduce energy waste, and improve yield efficiency. Elucidation of host‐pathway interactions establishes a sustainable framework for L‐TA production, advancing global green chemistry objectives.

## 2 Experimental Procedures

### 2.1 Strains, Plasmids, Media and Culture Conditions

The bacterial strains and plasmids constructed for this study are comprehensively documented in Table S1. Standard cultivation conditions were employed for each microbial strain: *E. coli* strains were maintained in LB medium or agar at 37°C, with kanamycin (50 μg/mL) or ampicillin (100 μg/mL) added as needed. *Citrobacter freundii* DSM14984 was propagated in Trypticase Soy Broth (Oxoid CM129) under standard conditions. For *G. oxydans* strains, cultivation was performed in mannitol medium (containing 5 g/L yeast extract, 3 g/L peptone, 25 g/L mannitol) at 30°C. To prevent contamination, cefoxitin (50 μg/mL) was added, taking advantage of the organism's natural antibiotic resistance profile. Initial isolation of strains was achieved by plating on MP agar, with individual colonies subsequently selected for biological replicate experiments. Pre‐cultures were prepared by inoculating 5 mL of medium in test tubes, followed by incubation at 30°C with shaking at 180 rpm. Scale‐up cultures were performed in 500 mL Erlenmeyer flasks containing 100 mL of MP medium under identical conditions. For large‐scale production, batch fermentations were conducted using CSLP medium, formulated with 0.41 g/L (NH_4_)_2_SO_4_, 0.1 g/L (NH_4_)_2_HPO_4_, 0.01 g/L MgSO_4_·7H_2_O, 3.0 g/L corn steep liquor paste, and 60–100 g/L glucose as the primary carbon source.

### 2.2 Molecular Cloning and Construction of Strains

Routine molecular biology procedures were performed using standard techniques as described in *Molecular Cloning: A Laboratory Manual* (Sambrook et al. 2014). For gene amplification, genomic DNA from *G. oxydans* ZJU2, *E. coli* K12, or *Citrobacter freundii* DSM14984 was used as a template to amplify the *P*_0169_, homologous fragments, and *gt* gene, respectively. Primer sequences for these amplifications are provided in Table S2. All DNA sequencing was performed by Sangon Biotech (Shanghai, China) to verify construct integrity. Plasmid transformation into *G. oxydans* strains was achieved through electroporation using optimised parameters (2000 V, 200 Ω, 25 μF), following our previous protocol (Yuan et al. 2016b).

Specifically, the *tktA*_M gene fragment (2004 bp) was PCR‐amplified from the pET28a(+)‐*tktA*_M plasmid and subsequently cloned into the *Xba* I/*Bam*H I‐digested pUCpr‐*P*_0169_ vector. This yielded the recombinant expression plasmid pUCpr‐*P*_0169_‐*tktA*_M, which was then transformed into *G. oxydans* ZJU2 via electroporation, generating the engineered *G. oxydans* T strain.

### 2.3 Protein Extraction and Quantification

For protein analysis, *G. oxydans* T cells from pre‐culture and scale‐up culture were harvested and disrupted using an ultrasonic homogeniser (JY96‐III BN, Xinzhi, Ningbo) with 30 pulse cycles (5 s on, 7 s off) maintained in an ice bath. Cell debris was then removed by centrifugation at 12000 × *g* for 15 min at 4°C to obtain clarified crude extracts. Protein concentration was determined using the Pierce BCA Assay Kit (ID: 23227, Thermo Scientific, China) according to the manufacturer's protocol, with bovine serum albumin as the standard.

### 2.4 Catalysis Condition and Enzyme Activity Measuring

To determine the optimal catalytic conditions for the engineered strain *G. oxydans* T strain, its oxidative activity was evaluated using 5‐KGA and glycolaldehyde as substrates. The pH profile was assessed across a broad range (pH 3.0–11.0) using an appropriate buffer system. Subsequent temperature optimization studies, conducted at this optimal pH, determined peak catalytic efficiency within the range of 20°C–50°C. Enzyme activity of the TKTA_M variant was quantified using an established pH‐based assay (Wang et al. 2022). In this study, one unit (1 U) of enzyme activity was defined as the amount of enzyme required to generate 1 μmol of product per minute under standard assay conditions. Specific activity (S, in U/mg) was calculated to normalise activity measurements to total protein content, using the following equation:

$$\begin{array}{cc} S\;\left(U/\mathrm{mg}\right) & =0.01085\times 10^{3}\;\left[\text{μmol}/\left(L\cdot s\right)\right]\times 60\;\left[s\right]\\ & \times 200\times 10^{-6}\;\left[L\right]/\text{enzyme amount}\;\left[\mathrm{mg}\right] \end{array}$$

where, 0.01085 represents the slope of the HCO_3_^−^ concentration change over time [μmol/(mL·s)], 60 defines the reaction time for 1 unit (U) of enzyme activity, in seconds (s), and 200 is the total reaction volume, in microliters (μL).

### 2.5 Preparation of *G. oxydans* T Resting Cells

The engineered *G. oxydans* T strain was cultured for whole‐cell biocatalyst preparation. Primary cultures were initiated by inoculating fresh MP medium followed by incubation at 30°C with orbital shaking (180 rpm) for 16 h to achieve logarithmic growth. Secondary cultures were established by transferring the primary culture (10% v/v inoculum) to fresh sterile medium under identical cultivation conditions for an additional 24 h to maximise cell biomass. For cell harvesting, cultures were centrifuged at 4000 × *g* for 20 min at 4°C. The resulting cell pellets were subsequently washed twice with ice‐cold 50 mM potassium phosphate buffer (pH 7.0) to remove residual medium components. Finally, the 90 g wet cells were resuspended in reaction assay buffer (50 mL total volume) containing 2.0 mM TEA buffer (pH 6.0), 60 g/L glucose, and 30 g/L glycolaldehyde.

### 2.6 Genomic Integration Expression

To enhance tartaric semialdehyde biosynthesis, we employed a pJKM vector mediated homologous recombination protocol to genetically modify *G. oxydans* . This approach involved two key genetic modifications: (1) overexpressing heterologous *tktA*_M (from *E. coli* K12) and *gt* (from *C. freundii* DSM 14984) genes, and (2) targeted knockout of three endogenous genes—*ga2dh* (GOX1231), *gdh*S (GOX2015), and *pdc* (GOX1081). The *tktA*_M overexpression cassette was constructed as follows: DNA fragments containing the *P*_0169_ promoter, *tktA*_M coding sequence, and homologous arms (U*gdh*S and D*gdh*S) were PCR‐amplified and purified. These fragments were then assembled with *Eco*R I and *Hind* III‐linearized pJKM vector using the pEASY‐Uni Seamless Cloning Kit (TransGen, Beijing, China), *yielding* pJKM‐U*gdh*S‐*P*_0169_‐*tktA*_M‐D*gdh*S. Successful construction was verified by DNA sequencing (Sangon Biotech, Shanghai). The recombinant plasmid was introduced into *G. oxydans* ZJU2 via electroporation, with successful integration into the *gdh*S locus confirmed by kanamycin resistance. Subsequent counter‐selection on 10% sucrose MP agar plate enabled the isolation of double‐crossover recombinants, resulting in marker‐free strain *G. oxydans* T01. Following the same strategy, we constructed plasmid pJKM‐U*pdc*‐*gt*‐D*pdc* and transformed it into *G. oxydans* T01 to generate the final engineered strain *G. oxydans* T02. This strain combines the *gt* gene integration at the *pdc* locus with the previously introduced *tktA*_M overexpression, creating an optimised pathway for tartaric semialdehyde accumulation.

### 2.7 Bioreactor‐Scale Production of Tartaric Semialdehyde

Tartaric semialdehyde was produced at bioreactor scale following an established bioconversion protocol (Hua et al. 2020) with modification. The fermentation was conducted in a 5.0 L bioreactor (working volume: 3.0 L) using CSLP medium supplemented with 100 g/L D‐glucose as carbon source, 15 g/L formaldehyde as co‐substrate, 0.85 g/L MgCl_2_ as cofactor, and 2% (v/v) ethanol as energy supplement. The engineered *G. oxydans* T02 strain served as the biocatalyst for this transformation. Process parameters were strictly controlled throughout the fermentation: temperature maintained at 30°C, agitation speed at 300 rpm, and aeration rate at 2 vvm. The pH was automatically regulated at 6.0 through the addition of 4.0 mol/L NaOH solution (Wang et al. 2022). Regular sampling was performed at predetermined intervals, and the samples were immediately analysed for substrate consumption and product formation according to previously described methods (Yuan et al. 2016a).

### 2.8 Analytical Methods

The concentration of GA, 5‐KGA and tartaric semialdehyde in the fermentation broth was quantitatively determined by HPLC. Analysis was performed using an RSpak DE‐613 analytical column (150 × 4.6 mm, Shodex, Japan) maintained at room temperature. The mobile phase consisted of 2 mM HClO_4_ delivered isocratically at a flow rate of 0.5 mL/min, with detection achieved by UV absorbance at 210 nm (Yuan et al. 2016a). Glucose concentration in the fermentation broth was monitored using a Bio‐Sensor (SBA‐40D, Shandong Academy of Sciences, China), which provided rapid measurements with a detection range of 0.1–100 g/L and ± 2% measurement accuracy. For biomass determination, cell dry weight was calculated from optical density measurements at 600 nm (OD_600_) using the following empirically derived correlation:

$$\;\text{Cell}\;\mathrm{dry}\;\text{weight}=0.35353\times {\mathrm{OD}}_{600}+0.12045,R^{2}=0.99979$$

where 0.35353 (slope) and 0.12045 (intercept) were derived from the linear regression of cell dry weight against optical density at 600 nm (OD_600_). R^2^ is the coefficient of determination.

### 2.9 Statistical Analysis

All experimental data were collected from three independent biological replicates to ensure reproducibility. Statistical analyses were performed using SPSS Statistics software (version 16.0, SPSS Inc., IBM Corporation, Chicago, IL, USA). Inter‐group comparisons were conducted using one‐way analysis of variance (ANOVA). Statistical significance was established at *p* < 0.05 for all analyses. Results are presented throughout as mean ± SD from triplicate experiments.

## 3 Results

### 3.1 Developing a Biosynthetic Pathway for Tartaric Semialdehyde in *G. oxydans*

The study commenced with genetic modification of *G. oxydans* ZJU2 (Yuan et al. 2016a, 2016b) strains to enhance 5‐KGA biosynthesis capacity. Building upon this foundation, we engineered a novel metabolic pathway by introducing a mutagenized version of the *E. coli* K12 *tktA* gene, which encodes the TKTA_M variant (R358I/H461S/R520Q). This engineered enzyme demonstrated catalytic activity in converting 5‐KGA to tartaric semialdehyde (Wang et al. 2022). The genetic construct was developed through PCR amplification of *tktA*_M, followed by its insertion into the pUCpr‐*P*_0169_ plasmid, yielding the recombinant plasmid pUCpr‐*P*_0169_‐*tktA*_M. Successful transformation of this constructed plasmid into *G. oxydans* ZJU2 generated the engineered strain *G. oxydans* T. Protein expression analysis via SDS‐PAGE confirmed the presence of a distinct ~72 kDa band, corresponding to the predicted molecular weight of TKTA_M (72.2 kDa), with solubility characteristics detailed in Figure S2.

**FIGURE 2:**
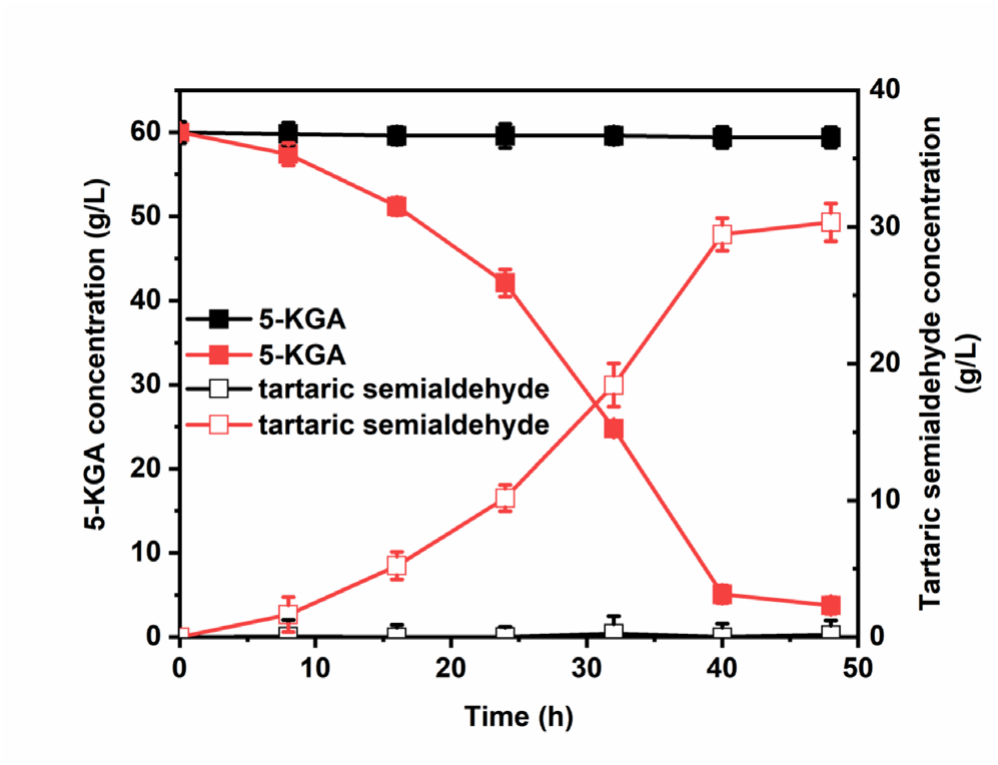
Time course of 5‐KGA consumption and tartaric semialdehyde production by *G. oxydans* strains. Symbols: ■, 5‐KGA, □, tartaric semialdehyde represents the control strain (ZJU2); 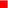, 5‐KGA and 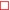, tartaric semialdehyde represents the engineered strain (T).

Functional characterisation revealed the engineered strain's remarkable bioconversion capability, achieving a tartaric semialdehyde titre of 30.35 ± 1.23 g/L with only 3.75 g/L residual 5‐KGA after 48 h cultivation (Figure 2). In contrast, the control strain exhibited minimal tartaric semialdehyde accumulation (< 0.5 g/L) and limited 5‐KGA consumption. The TKTA_M variant exhibited a specific activity of 3.72 ± 0.41 U/mg, consistent with previous reports (Wang et al. 2022), and demonstrated significant enhancement of the 5‐KGA conversion process compared to wild‐type strains. These results provide experimental validation for the hypothesised role of transketolase in L‐TA biosynthesis (Salusjärvi et al. 2004; Burbidge et al. 2021).

### 3.2 Biochemical Characterisation of *G. oxydans* T Strain Reactions

**FIGURE 3:**
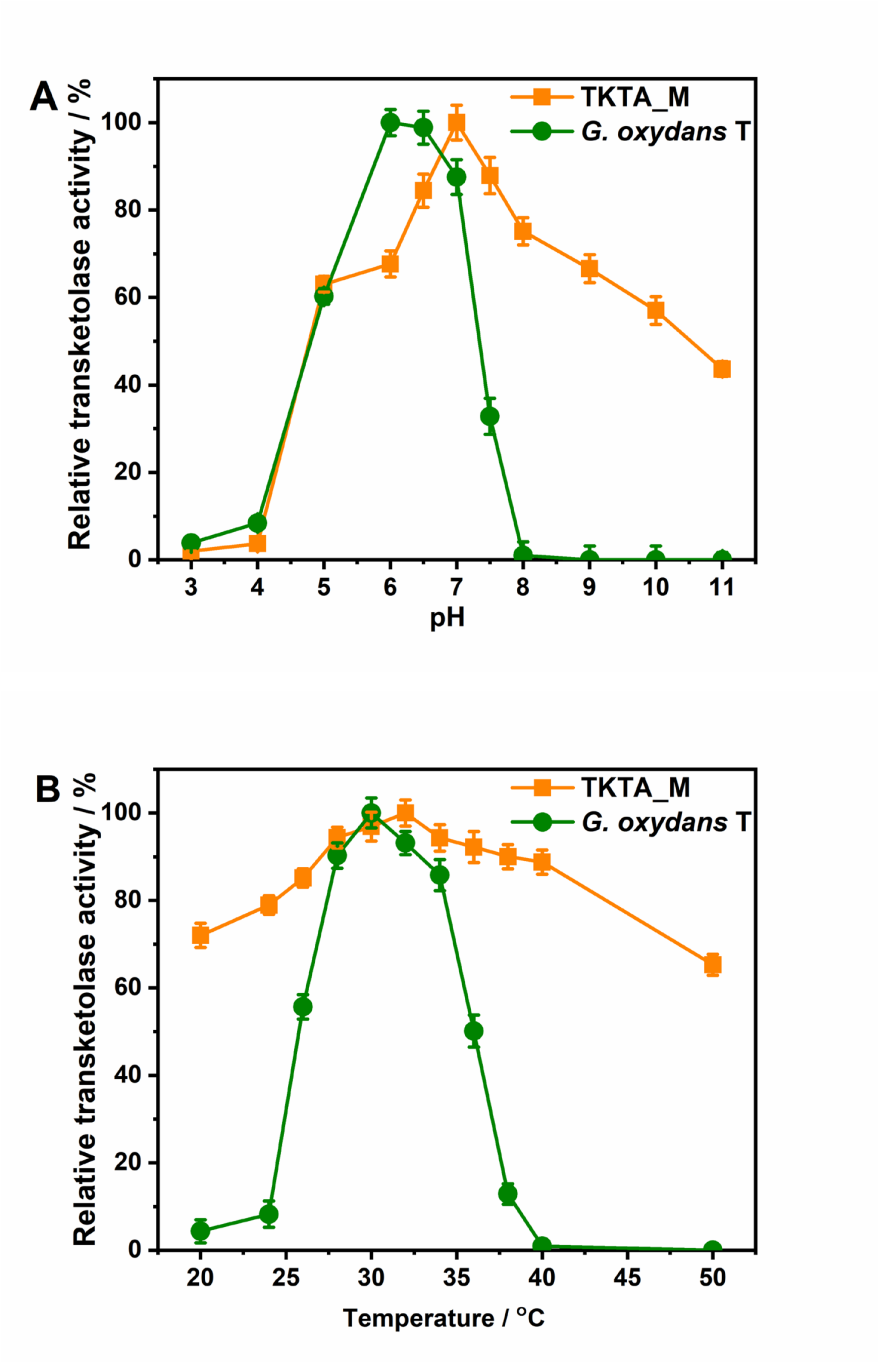
Effect of pH and temperature on the relative enzyme activity of pure TKTA_M and *G. oxydans* T. (A) optimal pH; (B) optimal temperature. Orange square (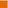) means pure TKTA_M enzyme; Green dark dot (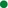) means engineered *G. oxydans* T strain.

We evaluated the catalytic performance of both purified TKTA_M enzyme and recombinant *G. oxydans* T whole‐cell biocatalyst across a range of pH and temperature conditions. Comparative analysis revealed distinct operational profiles between the isolated enzyme and whole‐cell systems (Figure 3). The whole‐cell biocatalyst exhibited maximum transketolase activity at pH 6.0, corresponding to the optimal growth conditions of the *G. oxydans* T strain (Yuan et al. 2016a). Notably, enzyme activity decreased sharply under alkaline conditions, with only 15%–20% residual activity at pH 7.0 and complete inactivation observed at pH > 8.0 (Figure 3A). In contrast, the purified TKTA_M demonstrated broader pH tolerance, showing peak activity at pH 7.0 with maintained functionality up to pH 9.0.

Temperature profiling identified an optimal operating range of 28°C–34°C for the whole‐cell system, with maximal activity occurring at 30°C (Figure 3B). Activity decreased precipitously at temperatures exceeding 34°C, reflecting the host organism's mesophilic nature. In contrast, the purified TKTA_M enzyme exhibited greater thermostability, maintaining 82.4% of maximal activity at 37°C and retaining 65.3% functionality at 50°C. For TKTA_M enzyme, thermal inactivation kinetics analysis revealed temperature‐dependent degradation constants (*K*_d_) of 0.058 h^−1^ at 40°C and 0.228 h^−1^ at 50°C, corresponding to functional half‐lives of 11.95 and 3.04 h, respectively (Wang et al. 2022). These data collectively demonstrate the enzyme's compromised structural stability at elevated temperatures.

### 3.3 Glucose Conversion in Engineered *G. oxydans* T Strains

To evaluate the effect of TKTA_M expression on metabolic flux redistribution, we performed comparative analyses of glucose oxidation pathways in the engineered *G. oxydans* T strain versus the *G. oxydans* ZJU2 strain under optimised culture conditions (pH 6.0, 30°C). HPLC‐based metabolite profiling revealed three distinct phases of bioconversion (Table 1).

1. Initial oxidation phase (0–8 h): both strains exhibited similar metabolic profiles, with rapid accumulation of gluconic acid (GA) as the primary oxidation product. The comparable GA production rates between strains indicated that the initial glucose oxidation machinery remained unaffected by genetic modifications.
2. Intermediate metabolic divergence (8–16 h): There were marked differences that emerged in 5‐KGA metabolism. *G. oxydans* ZJU2 produced 10.9 ± 0.27 g/L of 5‐KGA, while engineered *G. oxydans* T strain showed 27.5% lower 5‐KGA accumulation (7.9 ± 0.21 g/L), but the initiated tartaric semialdehyde production was 2.63 ± 0.34 g/L. This metabolic redistribution suggests the temporary metabolic burden from heterologous *tktA*_M overexpression (Yuan et al. 2016b). Notably, there is a competitive partitioning of carbon flux between native (5‐KGA) and engineered (tartaric semialdehyde) pathways.
3. Late‐stage bioconversion (16–48 h): The engineered *G. oxydans* T strain exhibited superior pathway performance, which achieved 32.21 ± 0.74 g/L tartaric semialdehyde titre compared to that of wild‐type *G. oxydans* ZJU2 strain (0.21 ± 0.13 g/L). It demonstrated a 153‐fold higher tartaric semialdehyde:5‐KGA ratio (2.33:1) compared to wild‐type *G. oxydans* ZJU2 (0.015:1). This observation suggests that *tktA*_M gene expression enhanced the tartaric semialdehyde pathway, thereby facilitating glucose conversion. However, the persistent 5‐KGA pool (13.83 ± 0.41 g/L) suggests the potential bottlenecks that are oxygen competition with membrane‐bound dehydrogenases (Yuan et al. 2016c) and imbalance between catalytic and synthesis efficiencies (Solovjeva et al. 2020). These findings demonstrate successful pathway engineering while highlighting opportunities for further optimization.

**TABLE 1:** Conversion of glucose to tartaric semialdehyde by *G. oxydans* T and *G. oxydans* ZJU2 cells (as control).

Time (h)	Products (g/L)					
	GA		5‐KGA		Tartaric semialdehyde	
	ZJU2	T	ZJU2	T	ZJU2	T
8	11.76 ± 0.12	10.93 ± 0.20	2.62 ± 0.31	0.82 ± 0.51	—	0.11 ± 0.01
16	37.74 ± 0.36	35.68 ± 0.12	10.90 ± 0.27	7.90 ± 0.21	—	2.63 ± 0.34
24	20.51 ± 0.13	23.42 ± 0.01	25.94 ± 0.36	21.32 ± 0.82	0.12 ± 0.11	6.91 ± 0.21
32	16.96 ± 0.23	17.67 ± 0.12	38.14 ± 0.13	23.46 ± 0.24	—	13.62 ± 0.83
40	13.76 ± 0.58	12.66 ± 0.17	44.58 ± 0.12	16.57 ± 0.34	0.21 ± 0.13	27.13 ± 1.02
48	11.61 ± 0.20	9.58 ± 0.43	46.65 ± 0.30	13.83 ± 0.41	0.20 ± 0.11	32.21 ± 0.74

Abbreviation: “—”: undetected.

### 3.4 Development of Marker‐Free Engineered Strains for Sustainable Bioproduction

**FIGURE 4:**
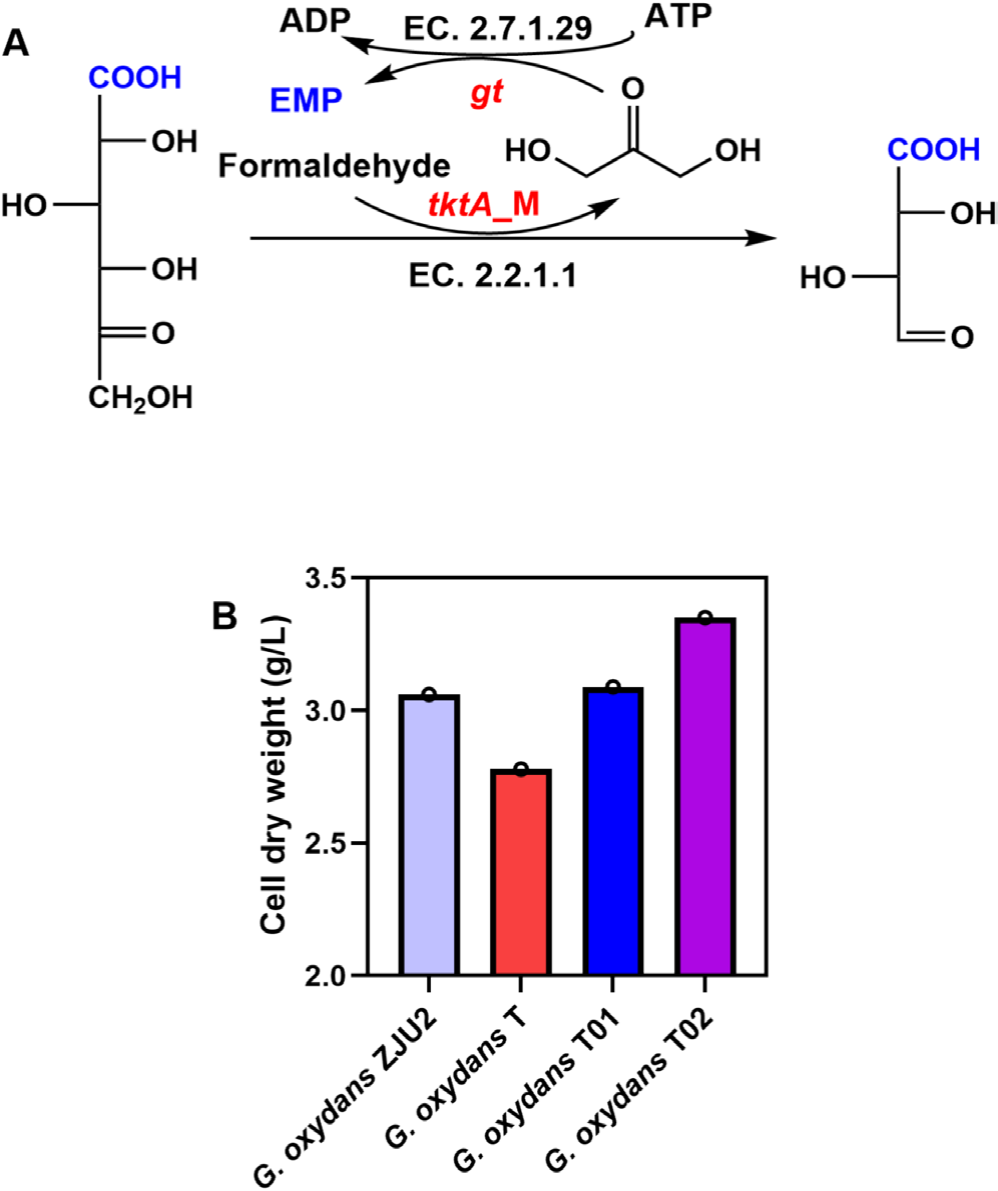
(A) Schematic representation of TKTA_M receptor coupling system construction, in which 5‐KGA was as donor and formaldehyde was as receptor. *tkt*A‐M, transketolase mutants; *gt*, glycerone kinase; EMP, Embden‐Meyerhof‐Parnas pathway; (B) Cell growth of each strain using glucose as sole carbon source.

The widespread use of antibiotic resistance genes as selection markers has raised scientific community concerns, particularly regarding horizontal gene transfer to pathogenic microorganisms, which poses significant risks to public health and ecosystems (Ramessar et al. 2007). To circumvent these issues, we employed a marker‐free genetic engineering strategy to construct *G. oxydans* T01 and T02 strains for tartaric semialdehyde production. These strains were developed using a “push‐pull” metabolic engineering approach combined with chromosomal integration of key genes. In this system, TKTA_M facilitated the transfer of a “ketol” group from 5‐KGA to an acceptor molecule, forming tartaric semialdehyde (Cárdenas‐Fernández et al. 2021; Solovjeva 2023). Furthermore, the heterologous *gt* gene from *C. freundii* DSM 14984 (Bächler et al. 2005) was introduced to convert formaldehyde into glycerone phosphate, which subsequently entered central carbon metabolism via the Embden‐Meyerhof pathway (Figure 4A).

Initial growth profiling revealed that the *G. oxydans* T strain, carrying plasmid‐based expression cassettes, exhibited impaired growth (~40% reduction in biomass) compared to wild‐type *G. oxydans* ZJU2 strain when cultured in 60 g/L glucose medium for 48 h (Figure 4B). This growth defect likely stemmed from metabolic stress induced by high‐level heterologous gene expression under the strong *P*_0169_ promoter and the physiological burden of antibiotic application (Ryu et al. 2017).

In contrast, the chromosomally integrated *G. oxydans* T01 strain exhibited growth kinetics comparable to the wild‐type control, whereas the *G. oxydans* T02 strain (carrying both *tktA*_M and *gt* integrations) showed a 23.85% increase in final biomass density. This enhanced growth phenotype results from three main factors: stable genomic integration reducing metabolic burden (Hao et al. 2021), improved metabolic efficiency through the *gt*‐mediated carbon assimilation pathway, and marker‐free selection eliminating antibiotic‐induced stress (Saleski et al. 2021). These results validate our metabolic engineering strategy, demonstrating that chromosomal integration of the *gt* gene coupled with optimised pathway expression can simultaneously improve product formation and host fitness. The *G. oxydans* T02 strain was consequently selected for subsequent bioprocess studies.

### 3.5 Scale‐Up Production of Tartaric Semialdehyde With Engineered *G. oxydans* T02

**FIGURE 5:**
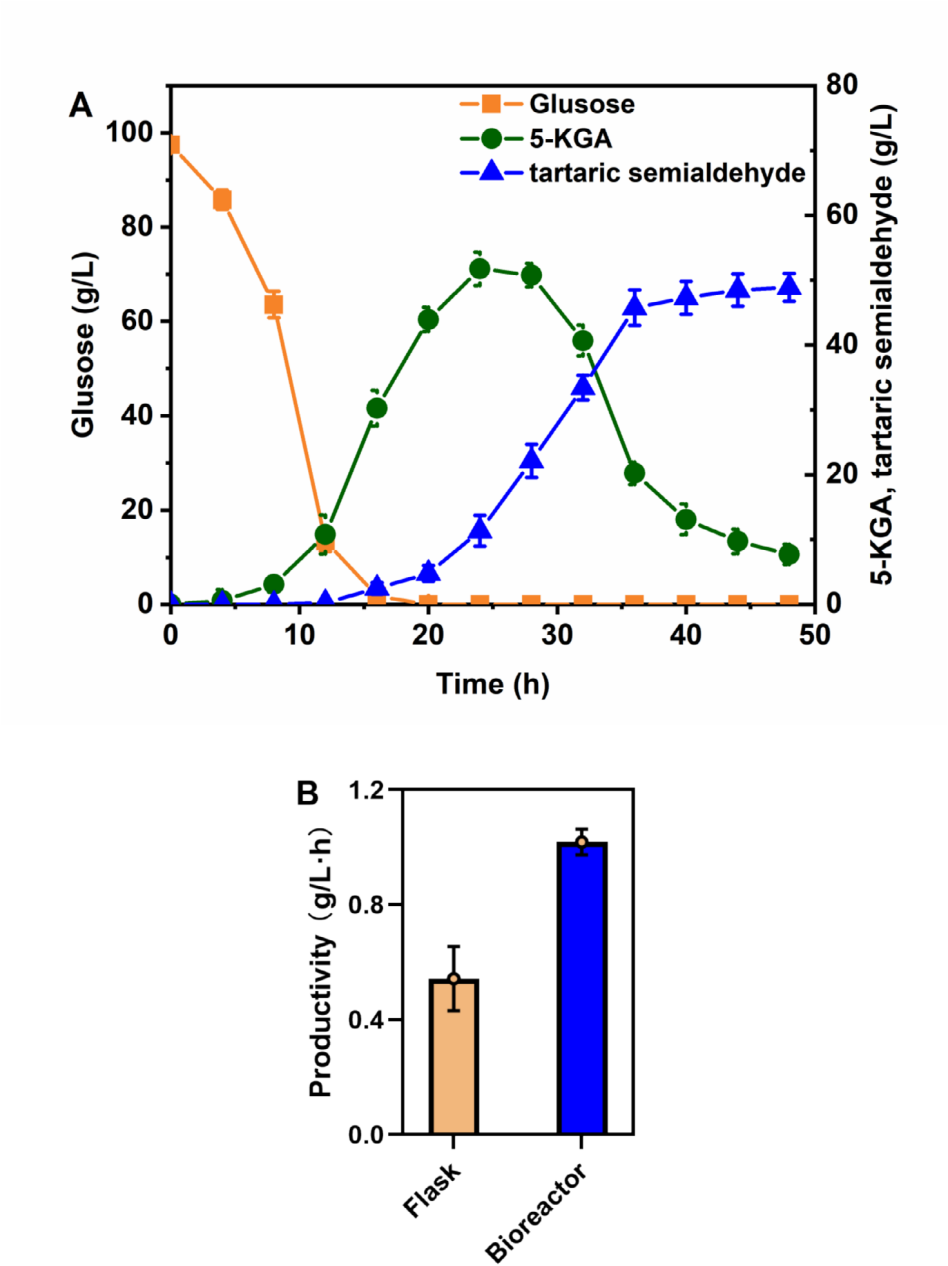
Batch fermentation performance of the *G. oxydans* T02 strain using 100 g/L glucose as substrate for tartaric semialdehyde production in shake flask and a 5‐L bioreactor. (A) The temporal evolution of glucose (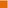 ), 5‐KGA (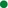 ), and tartaric semialdehyde (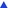 ) concentrations in the 5‐L bioreactor. (B) The productivity of tartaric semialdehyde in shake flask and a 5‐L bioreactor.

To assess the industrial potential of engineered strain, the scale‐up fermentation of *G. oxydans* T02 was conducted in a 5 L bioreactor using glucose (100 g/L) as the sole carbon source in CSLP medium. The batch fermentation process yielded 48.88 ± 2.16 g/L tartaric semialdehyde within 48 h, with 7.72 ± 1.56 g/L residual 5‐KGA remaining (Figure 5A), demonstrating efficient carbon flux through the engineered pathway. In line with previous studies on *G. oxydans* (Richhardt et al. 2013; Yuan et al. 2016a), we observed a biphasic metabolism pattern: a rapid oxidation phase (0–16 h) where approximately 90% of glucose was converted to GA, and a product formation phase (16–48 h) with tartaric semialdehyde accumulating at 1.018 g/L·h, an 87.82% increase over flask‐scale cultivation (Figure 5B). Compared to previous 5‐KGA production studies (Yuan et al. 2016a, 2016b), the tartaric semialdehyde productivity was 40%–48% of the 5‐KGA benchmark (2.10–2.53 g/L·h). While conversion efficiency needs optimisation, and this study marks the first *de novo* production of tartaric semialdehyde from glucose, successful scale‐up to bioreactor cultivationlays the groundwork for cost‐effective biomanufacturing.

## 4 Discussion

Advancements in synthetic biology now allow biotechnological tools to produce various non‐fossil fuels and chemicals by creating novel biosynthetic pathways (Ramos and Segura 2024). In this study, the successful development of engineered *G. oxydans* strains for tartaric semialdehyde production represents a significant advancement in microbial biosynthesis of tartaric acid precursors. Our findings demonstrate several key innovations in metabolic engineering and provide important insights into the physiological constraints of *G. oxydans* as a biocatalyst.

### 4.1 Catalytic Performance of Engineered Strains

Transketolase, a thiamine diphosphate (ThDP) and Mg^2+^ dependent enzyme, facilitates stereoselective carbon–carbon bond formation in ketose synthesis (Chu et al. 2023; Ocal et al. 2023). Its activity with nonphosphorylated polyhydroxylated aldehyde acceptors has previously been investigated in wild‐type enzymes from *E. coli* , yeast, or spinach (Ranoux et al. 2012). While directed evolution can enhance its activity with these substrates (Hibbert et al. 2007), further research on reaction conditions is needed.

The recombinant TKTA_M enzyme exhibited distinct catalytic properties in *G. oxydans* compared to previous reports that were from *E. coli* systems (Wang et al. 2022). Notably, the optimal activity at pH 6.0 and 30°C reflects adaptation to the native physiological conditions of *G. oxydans* , differing from those reported for other microbial systems (pH 7.0–8.0, 25°C–37°C) (Zhou 2016). This host‐specific enzyme behaviour underscores the importance of considering microbial physiology when engineering heterologous pathways. The 3.72 ± 0.41 U/mg specific activity achieved in whole‐cell catalysis demonstrates efficient functional expression of TKTA_M, while the observed thermal instability above 40°C (*K*_d_ = 0.228 h^−1^) informs practical limitations for industrial application.

### 4.2 Metabolic Engineering Strategy Effectiveness

The recombinant *G. oxydans* T strain showed improved bioconversion efficiency over the wild‐type, confirming that genetic modifications can enhance the conversion of 5‐KGA to tartaric semialdehyde, consistent with the L‐TA biosynthesis pathway involving transketolase and succinate semialdehyde dehydrogenase (Salusjärvi et al. 2004), while advancing the field through the implementation of a novel “Push‐Pull” strategy. This dual strategy (Sun et al. 2023) yielded several significant outcomes. The chromosomal integration strategy effectively eliminated antibiotic markers, thereby addressing biosafety concerns (Ramessar et al. 2007) while enhancing strain stability. Notably, *G. oxydans* T02 exhibited a 23.85% increase in growth compared to the wild‐type, defying the typical metabolic burden anticipated from heterologous expression. At the bioreactor scale, production achieved 48.88 ± 2.16 g/L of tartaric semialdehyde with a productivity rate of 1.018 g/L·h, demonstrating scalability. These findings validate the dual strategy of enhancing the supply of 5‐KGA through native pathway engineering (“Push”) and directing carbon flux toward tartaric semialdehyde via gt integration (“Pull”).

### 4.3 Physiological Considerations and Limitations

Despite the successful application of the “Push‐Pull” strategy, several metabolic constraints have been identified. The incomplete phosphotransferase system (PTS), lacking EIIC and EIIB components (Prust et al. 2005), limits the efficiency of glucose uptake. Additionally, the absence of certain TCA cycle enzymes restricts energy generation (Bringer and Bott 2016), leading to low cellular biomass. Competing oxidation reactions further diminish the carbon yield for the desired products. Notably, the persistence of 13.83 ± 0.41 g/L residual 5‐KGA indicates potential issues such as insufficient TKTA_M activity for complete substrate conversion, diversion of carbon by competing metabolic fluxes away from the engineered pathway, or potential transport limitations affecting intracellular access to 5‐KGA.

These limitations indicate three primary areas for future optimisation: (1) pathway optimisation, which involves the precise modulation of expression levels of TKTA_M and auxiliary enzymes; (2) redox engineering, aimed at balancing cofactor requirements to enhance efficiency; and (3) process development, which focuses on optimising aeration and feeding strategies to mitigate oxygen sensitivity.

## 5 Conclusion

Currently, no studies have documented the synthesis of L‐TA using transketolase with renewable resources. This research establishes *G. oxydans* as a feasible platform for tartaric semialdehyde production through innovative metabolic engineering techniques. The implementation of a “Push‐Pull” strategy successfully redirected carbon flux without compromising cell viability, offering a framework for the genetic engineering of this organism. Future investigations that address the identified physiological limitations could facilitate the commercially viable production of tartaric acid precursors from renewable feedstocks.

## Author Contributions

**Shuangxi Li:** writing – original draft, data curation, methodology. **Lingcheng Li:** investigation, resources, performance. **Qiwu Jiang:** investigation, performance. **Jianfeng Wang:** writing – review and editing, resources. **Xiaoming Sun:** supervision, resources, funding acquisition. **Liangliang Zhang:** writing – review and editing. **Jianfeng Yuan:** conceptualisation, methodology, supervision, resources.

## Conflicts of Interest

The authors declare no conflicts of interest.

## Supporting information


Data S1.

## Data Availability

The data that support the findings of this study are available from the corresponding author upon reasonable request.
