# Supplementary material for: From Glucose to Green Chemistry: Breakthrough in Microbial Production of Tartaric Semialdehyde
Source: Microb Biotechnol. 2025 Apr 23;18(4):e70149. doi: 10.1111/1751-7915.70149 (PMC12016104; doi:10.1111/1751-7915.70149)
Supplement: Supplementary file 1 — Data S1. [file MBT2-18-e70149-s001.docx]

Additional file

Table S1: Strains and plasmids used in this study

| - **Strains** | - **Relevant characteristics** | - **Source** |
| --- | --- | --- |
| - *E. coli* DH5α | - F-Φ80*lacZ*ΔM15 Δ(*lacZYA-argF*) U169 *endA1 recA1* *hsdR17* (rK^-^, mK^+^) *supE44* *thi-1 gyrA96 relA1 phoA* | - Invitrogen |
| - *E. coli* K-12 | - Wild-type (WT) | - Invitrogen |
| - *Citrobacter freundii DSM14984* | - Wild-type (WT) | - DSMZ |
| - *G. oxydans* ZJU2 | - Gluconate 2-dehydrogenase and pyruvate decarboxylase deletion strain derived from *G. oxydans* DSM2343, Cef^R^ | - (Yuan JF, et al., 2016) |
| - *G. oxydans* T | - *G. oxydans* ZJU2 harboring pUCpr-*P_0169_*-*tkt*A_M, Cef^R^, Amp^R^ | - This work |
| - *G. oxydans* T01 | - *G. oxydans* ZJU2 Δ*gdh*S::*P_0169_*-*tkt*A_M, mutant TKTA_M derived from *tkt*A gene in *E. coli* K12 with promoter *P_0169_* integrated into the *gdh*S (GOX2015), Cef^R^ | - This work |
| - *G. oxydans* T02 | - *G. oxydans* T01 Δ*pdc*::*P_0169_*-*gt*, *gt* gene encoding glycerone kinase from *Citrobacter freundii* DSM 14984 with promoter *P_0169_* integrated into the *pdc* (GOX1081), Cef^R^ | - This work |
| - **Plasmids** |  |  |
| - pJKM | - Mobilized suicide vector derived from pK18mobGII, in which the *gus*A gene was replaced by *sac*B gene, to construct insertion or deletion mutants of *G. oxydans*, Kan^R^ | - (Yuan JF, 2016) |
| - pUCpr-*P_0169_* | - *P_0169_* promoter inserted in pUCpr (5132 bp), Amp^R^ | - (Yuan JF, 2016) |
| - pET28a(+)-*tkt*A_M | - *tkt*A_M gene expression vector in *E. coli* derived from pET28a(+), Kan^R^ | - (Wang JF, et al., 2022) |
| - pUCpr-*P_0169_*-*tkt*A_M | - *tkt*A_M expression vector in *G. oxydans*, Amp^R^ | - This work |
| - pJKM-U*gdh*S-*P_016_*_9_-*tkt*A_M-D*gdh*S | - plasmid for genomic integration of *P_0169_*-*tkt*A_M into the *gdh*S (GOX2015) locus of the genome of *G. oxydans* ZJU2, Kan^R^ | - This work |
| pJKM-U*pdc*-*gt*-D*pdc* | plasmid for genomic integration of *gt* gene into the *pdc* (GOX1081) locus of the genome of *G. oxydans* T01, Kan^R^ | This work |

| **No.** | **Primer** | **Purpose** | **Sequence (5’ → 3’)** |
| --- | --- | --- | --- |
| 1 | TKTA_*Xba*I_F | Amplification of *TKTA_M* gene from pET28a(+)-*TKTA_M* plasmid & sequencing | GCTCTAGAatgtcctcacgtaaag |
| 2 | TKTA_*BamH*I_R | Amplification of *TKTA_M* gene from pET28a(+)-*TKTA_M* plasmid & sequencing | CGGGATCCttacagcagttcttttgc |
| 3 | P_U*gdh*S_F | Amplification of the upstream flank of *gdh*S, and the 5’ -end overlaps with pJKM plasmid | *GCTATGACATGATTACG*tcccgctggccaagg |
| 4 | 0169_U*gdh*S_R | Amplification of the upstream flank of *gdh*S, and the 3’ -end overlaps with *P_0169_* fragment | *CGCGCCAGCCGCTTTCA*ctggcctcacctctcc |
| 5 | U*gdh*S_0169_F | Amplification of *P_0169_*, and the 5’ -end overlaps with U*gdh*S fragment | *GGAGAGGTGAGGCCAG*tgaaagcggctggcgcg |
| 6 | *tkt*_0169_R | Amplification of *P_0169_*, and the 3’ -end overlaps with *TKTA_M* fragment | *CTTTACGTGAGGACAT*gcggaaggcgttatac |
| 7 | 0169_*tkt*_F | Amplification of *TKTA_M*, and the 5’ -end overlaps with *P_0169_* fragment | *GTATAACGCCTTCCGC*atgtcctcacgtaaag |
| 8 | D*gdh*S_*tkt*_R | Amplification of *TKTA_M*, and the 3’ -end overlaps with D*gdh*S fragment | *AGAGGGGCCATATAAGT*ttacagcagttcttttg |
| 9 | *tkt*_D*gdh*S_F | Amplification of the downstream flank of *gdh*S, and the 5’ -end overlaps with *TKTA_M* fragment | *AATGTCGTCAAGAAAAC*acttatatggccccttc |
| 10 | P_D*gdh*S_R | Amplification of the downstream flank of *gdh*S, and the 3’ -end overlaps with pJKM plasmid | *GACGTCCGTACGTTCGA*ggccaggcggctggctg |
| 11 | *gdh*S_validation_F | Colony PCR Validation of insertion into *gdh*S (GOX2015); sequencing | ccgtcatcgagatgatcc |
| 12 | *gdh*S_validation_R | Colony PCR Validation of insertion into *gdh*S (GOX2015); sequencing | gggatttcagtcgtatcg |
| 13 | P_U*pdc*_F | Amplification of the upstream flank of *pdc*, and the 5’ -end overlaps with pJKM plasmid | *GCTATGACATGATTACG*ccggctgtattcaattc |
| 14 | *gt*_U*pdc*_R | Amplification of the upstream flank of *pdc*, and the 3’ -end overlaps with *gt* fragment | *CATTGATCAATTTTTTCAT*gatttcagtacctcagg |
| 15 | U*pdc*_*gt*_F | Amplification of  *gt*, and the 5’ -end overlaps with U*pdc* fragment | *CCTGAGGTACTGAAATC*atgaaaaaattgatcaatg |
| 16 | D*pdc*_*gt*_R | Amplification of  *gt*, and the 3’ -end overlaps with D*pdc* fragment | *GGATCAGACGCTTTGT*ttaaccctgacggttg |
| 17 | *gt*_D*pdc*_F | Amplification of the downstream flank of *pdc*, and the 5’ -end overlaps with *gt* fragment | *CAACCGTCAGGGTTAA*acaaagcgtctgatcc |
| 18 | P_D*pdc*_R | Amplification of the downstream flank of *pdc*, and the 3’ -end overlaps with pJKM plasmid | *GACGTCCGTACGTTCGA*gggtagcattgtcggtaagg |
| 19 | *pdc*_validation_F | Colony PCR Validation of insertion into *pdc* (GOX1081); sequencing | tctggatccggaacatcagg |
| 20 | *pdc*_validation_R | Colony PCR Validation of insertion into *pdc* (GOX1081); sequencing | atgtcatctagaggttcttttc |

Table S2: Oligonucleotides used in this study. Restriction restriction sites are underlined.

Scheme 1.


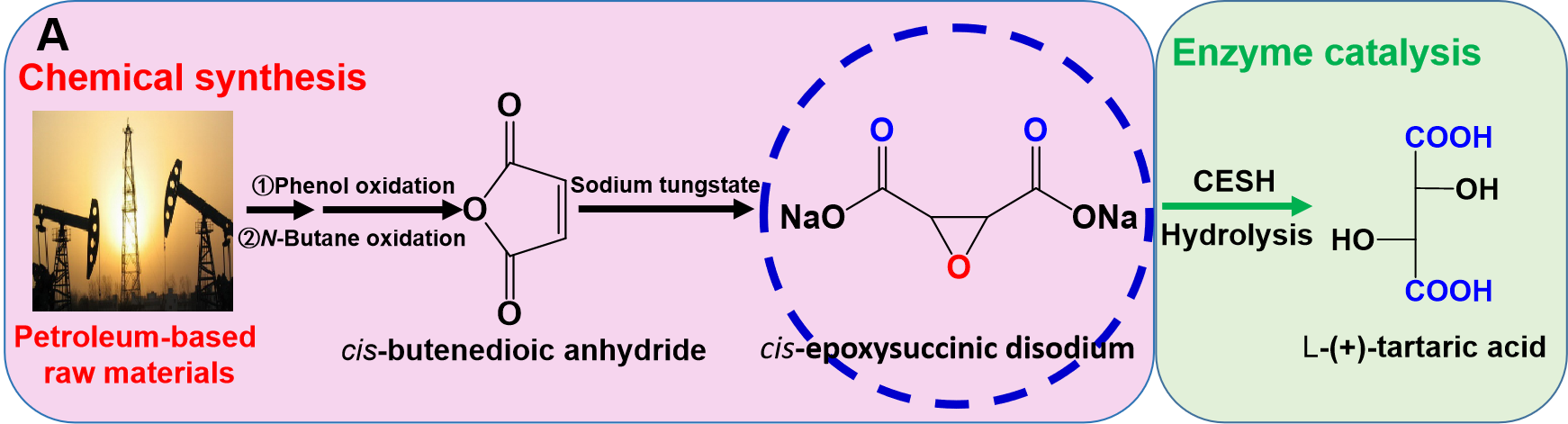

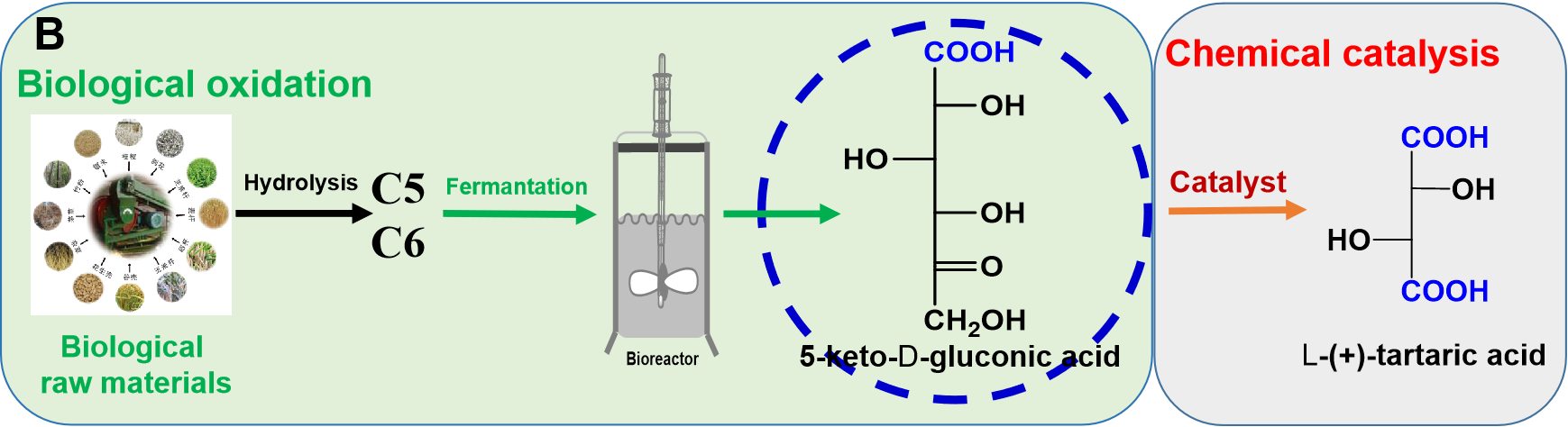


**Scheme 1.** Two stage preparation of L-(+)-tartaric acid by (**A**) conventional enzyme catalysis method by hydrolyzed the *cis*-epoxysuccinic disodium obtained from petroleum-based raw materials, and (**B**) chemical catalyzed 5-keto-D-gluconic acid (5-KGA) via biological raw materials. CESH, *cis*-epoxysuccinate hydrolase (EC 3.3.2.3).


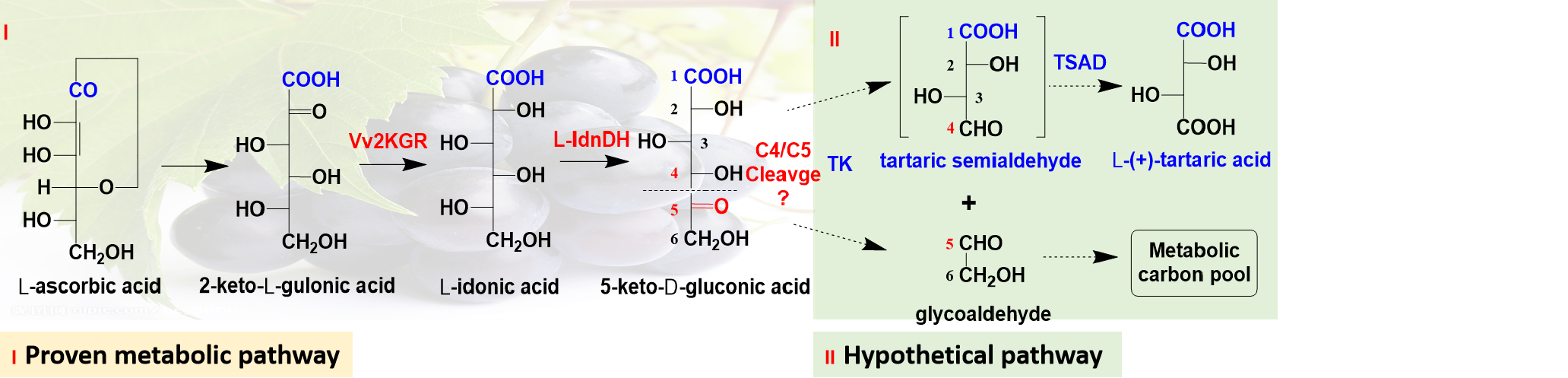


**Figure S1.** L-TA biosynthesis pathway in *Vitis vinifera* modified according to the literature. Solid arrow present the enzymes identified. dashed arrow means that the key enzymes have not been identified yet. Vv2KGR, 2-keto-L-gulonic acid reductase; L-IdnDH, L-idonic acid dehydrogenase; TK, hypothetical transketolase; TSAD, hypothetical tartaric acid semialdehyde dehydrogenase.


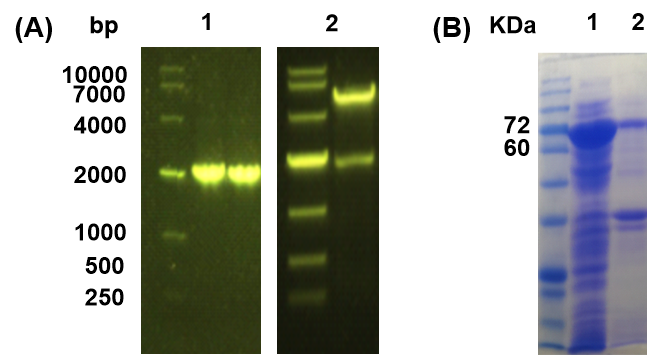


Figure S2: Clone and expression of *TKTA_M* gene from pET28a(+)-*TKTA_M*, which constructed before. (A) The *TKTA_M* gene clone and identification. Lane 1, PCR amplification of *TKTA_M* gene, Lane 2, double enzyme digestion to verify the pUCpr-*P_0169_*-*TKTA_M*; (B) The *TKTA_M* enzyme expression in engineered *G. oxydans* T. Lane 1 soluble expression, Lane 2 corresponding insoluble expression.

**Reference**

Wang JF, Li WY, Xin ZQ, Feng WN, Sun XM, Yuan JF. Molecular engineering of transketolase from *Escherichia coli* and tartaric semialdehyde biosynthesis. Chin J Biotech. 2022; 38: 4615-4629.

Yuan JF. Manufacture of L-(+)-tartaric acid by sequential whole-cell oxidation and chemical catalysis. PhD thesis. Zhejiang University, College of Chemical and Biological Engineering; 2016.

Yuan JF, Wu MB, Lin JP, Yang LR. Enhancement of 5-keto-d-gluconate production by a recombinant *Gluconobacter oxydans* using a dissolved oxygen control strategy. J Biosci Bioeng. 2016; 122:10-16.
